# Adipocyte Fatty-Acid Binding Protein is Overexpressed in Cirrhosis and Correlates with Clinical Outcomes

**DOI:** 10.1038/s41598-017-01709-0

**Published:** 2017-05-12

**Authors:** Isabel Graupera, Mar Coll, Elisa Pose, Chiara Elia, Salvatore Piano, Elsa Solà, Delia Blaya, Patricia Huelin, Cristina Solé, Rebeca Moreira, Gloria de Prada, Núria Fabrellas, Adrià Juanola, Manuel Morales-Ruiz, Pau Sancho-Bru, Càndid Villanueva, Pere Ginès

**Affiliations:** 1 Liver Unit, Hospital Clinic, University of Barcelona, Barcelona, Spain; 2 Institut d’Investigacions Biomèdiques August Pi I Sunyer (IDIBAPS), Barcelona, Spain; 3 Centro de Investigación Biomédica en Red de Enfermedades Hepáticas y Digestivas (CIBEREHD), Barcelona, Spain; 4 School of Medicine and Health Sciences Center, University of Barcelona, Barcelona, Spain; 5 Biochemistry and Molecular Genetics Department. Hospital Clínic, Department of Biomedicine-Biochemistry Unit, School of Medicine University of Barcelona, Barcelona, Spain; 6 Gastroenterology department, Hospital de la Santa Creu i Sant Pau, Barcelona, Spain

## Abstract

Fatty-acid-binding proteins (FABPs) are small intracellular proteins that coordinate lipid-mediated processes by targeting metabolic and immune response pathways. The aim of the study was to investigate plasma FABPs levels and their relationship with clinical outcomes in cirrhosis. Plasma levels of L-FABP1(liver and kidney), I-FABP2(intestine), and A-FABP4(adipocyte and macrophages) were measured in 274 patients with decompensated cirrhosis. Hepatic gene expression of FABPs was assessed in liver biopsies from patients with decompensated cirrhosis and in liver cell types from mice with cirrhosis. Immunohistochemistry of A-FABP4 in human liver biopsy was also performed. Plasma levels of FABPs were increased in patients with decompensated cirrhosis compared to those of healthy subjects (L-FABP1: 25 (17–39) vs 10 (9–17) ng/mL p = 0.001, I-FABP2: 1.1 (0.5–2.1) vs 0.6 (0.4–1) ng/mL p = 0.04 and A-FABP4: 37 (20–68) vs 16 (11–33) ng/mL p = 0.002), respectively. Increased A-FABP4 levels were associated with complications of cirrhosis, acute-on-chronic liver failure and poor survival. Hepatic A-FABP4 gene expression was upregulated in decompensated cirrhosis. Macrophages were the main liver cell that over-expressed A-FABP4 in experimental cirrhosis and increased A-FABP4 was found in macrophages of human biopsies by immunohistochemistry. A-FABP4 levels are increased in decompensated cirrhosis and correlate with poor outcomes. Liver macrophages appear to be the main source of A-FABP4 in decompensated cirrhosis.

## Introduction

Fatty-acid-binding proteins are small intracellular proteins of 14–15 KDa expressed in several tissues that coordinate lipid-mediated processes in cells by targeting metabolic and immune response pathways. At least 9 types of FAPBs have been identified and they are named depending on the organ or tissue where they were discovered or are prominently expressed (liver, intestine, heart, fat…etc.)^1^. FABPs share a characteristic three-dimensional configuration characterized by 10-stranded antiparallel β3-barrel structure with a fatty acid-binding pocket located inside its β-barrel. FABPs facilitate the transport of fatty acids to specific cell compartments where they exert their biological functions including, among others, membrane synthesis, oxidation, regulation of enzyme activity, and lipid-mediated transcriptional regulation.

Although FABPs were initially described as intracellular chaperones primary involved in lipid metabolism, FAPBs effects are different according to tissues or cell types. The delivery of fatty acids to certain intracellular compartments in a specific tissue or cell leads to different protein-protein and protein-membrane interactions, which trigger functions that are tissue characteristic. Liver fatty-acid binding protein 1 (L-FABP1) is highly abundant in the liver but is also expressed in intestine, pancreas, kidney, lung, and stomach. L-FABP1 is the only FABP that can bind two long-chain fatty acids at the same time. Although the specific function of L-FABP1 in the liver is not completely known, it has been suggested that L-FABP1 would mainly act as a long-chain fatty acid transporter targeting the ligands to β-oxidation pathways^2^. Intestinal fatty-acid binding protein 2 (I-FABP2), is manly expressed in the epithelium of small intestine and contributes to lipid absorption and metabolism^1, 2^. Adipocyte fatty-acid binding protein (A-FABP4) is mainly expressed in adipocytes and macrophages and regulates adipocyte fatty-acid uptake and lipogenesis and delivery of lipids to nuclear receptors mediating nuclear transcriptional programs. Interestingly, in macrophages A-FABP4 modulates inflammatory responses and cholesterol ester accumulation^2^. Specific actions of other FABPs are discussed elsewhere^1^.

Besides its intracellular specific cell functions, FABPs are released into the circulation and increased plasma levels of different FABPs have been found in several clinical conditions and have been proposed as markers of tissue injury^1, 3, 4^. For example, L-FABP1 plasma levels are increased in patients with acute rejection after liver transplantation^5^; plasma levels of I-FABP2 are increased in intestinal ischemia and are a marker of intestinal epithelium damage and sepsis of abdominal origin^6, 7^; heart and brain FABPs (H-FABP3 and B-FABP7) are released into the circulation immediately after cardiac or brain cell damage^4^; plasma A-FABP4 levels are increased in several metabolic (obesity, type-2 diabetes) and cardiovascular conditions (arterial hypertension, cardiac dysfunction and atherosclerosis) and have been shown to predict long-term cardiovascular events^3, 8, 9^. Furthermore, A-FABP4 plasma levels are increased in critically-ill patients and correlate with poor prognosis, which suggests that A-FABP4 is not only a marker of metabolic syndrome but also an inflammatory marker of poor outcome^10^.

Advanced cirrhosis is characterized not only by alterations in liver function, but also by abnormalities in many other organs including the gut and the immune system. Liver inflammation causes release of damage-associated molecular patterns (DAMPs). Moreover, intense alterations in the intestinal barrier, secondary to portal hypertension, lead to bacterial translocation and release of pathogen-associated molecular patterns (PAMPs). Both, DAMPs and PAMPs activate the immune system causing a persistent low-grade systemic inflammation that may contribute to cirrhosis progression, disease decompensation and development of acute-on-chronic liver failure (ACLF) syndrome^11–14^.

Although the liver plays an important role in lipid metabolism, little is known about FABPs in cirrhosis. Hepatic gene expression of L-FABP1 has been shown to be down-regulated in non-alcoholic fatty liver disease (NAFLD)^15^ and A-FABP4 plasma levels have been associated with liver inflammation in NAFLD patients^16^. Because advanced cirrhosis is characterized by abnormalities in different organs (liver, intestine, kidney, immune system…), we hypothesized that cirrhosis could be associated with alterations in FABPs plasma levels that could correlate with complications and disease outcomes. On this background, the aim of the present study was to investigate FABPs plasma levels and hepatic gene expression in decompensated cirrhosis and assess the relationship between FABPs and outcomes in cirrhosis. We studied three FABPs because of their potential pathogenic interest in cirrhosis: L-FABP-1 and I-FABP2 because of their role in liver and intestinal damage and A-FABP4 because of its relationship with systemic inflammation and immune system.

## Results

### Characteristics of the patient population

274 patients admitted to the hospital for complications of cirrhosis were included in this study. Demographic and clinical characteristics of the cohort are shown in Table 1. Patients had impaired liver function as reflected by increased serum bilirubin and INR and decreased serum albumin, with median MELD and Child-Pugh scores of 18 (IQ range:13–25) and 9 (IQ range:8–11), respectively. More than two thirds of patients had had previous complications of cirrhosis before the index hospitalization. At admission 183 patients (67%) had ascites, 126 (47%) acute kidney injury, 101 (37%) infection, 86 (31%) hepatic encephalopathy and 46 (18%) gastrointestinal bleeding. Sixty patients (19%) were admitted to the ICU, whereas the remaining patients were admitted to a regular ward.

**Table 1:** Demographic, clinical and laboratory data of the 274 patients at inclusion.

Variable	
Age (yr)	58 (52–67)
Gender (Male)	177 (65)
Etiology of cirrhosis. Alcoholic/Hepatitis C/Other	149/115/10
Previous complications of cirrhosis*	210 (76)
Chronic kidney disease**	45 (16)
Presence of ascites	183 (67)
Presence of encephalopathy	86 (31)
Norfloxacin prophylaxis	62 (23)
Antibiotic treatment	158 (59)
Treatment with beta-blockers	52 (19)
Serum bilirubin (mg/dL)	2.4 (1.3–5.1)
Serum albumin (g/L)	28 (25–32)
INR	1.5 (1.3–1.9)
Serum creatinine (mg/dL)	1.1 (0.8–1.9)
Serum sodium (mEq/L)	135 (131–137)
Blood leukocytes (10^9^/L)	5.7 (3.8–8.8)
C-reactive protein (mg/dL)	2.3 (0.9–5.0)
IL-6 (pg/mL)	110 (48–208)
Mean arterial pressure (mmHg)	83 (75–90)
MELD score	18 (13–25)
Child-Pugh score	9 (8–11)

Categorical variables are expressed as numbers and percentages (in brackets), continuous variables are expressed as median (inter-quartile range). MELD, model of end stage liver disease; *Ascites/Hepatic Encephalopathy/GI bleeding/SBP: 188/88/69/23, respectively **Parenchymal nephropathy in 26 patients and type-2 HRS in 24 patients, unknown in 2 patients.

### Plasma FABPs levels and their relationship with complications of cirrhosis and ACLF

**Figure 1:**
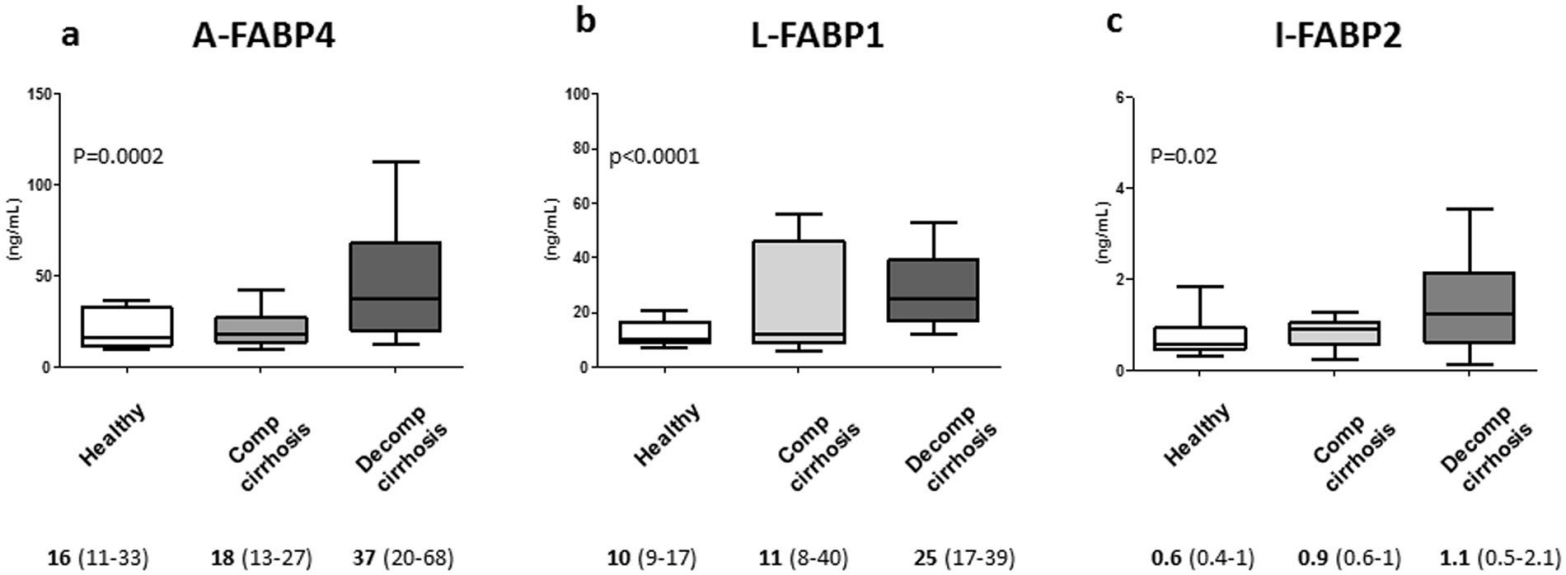
Plasma levels of FABPs in patients with decompensated cirrhosis compared to patients with compensated cirrhosis and healthy subjects. Panel a shows plasma levels of adipocyte fatty-acid binding protein 4 (A-FABP4), panel b shows plasma levels of liver fatty-acid binding protein 1 (L-FABP1), and panel c shows plasma levels of intestinal fatty-acid binding protein 2 (I-FABP2). Values shown below the panels are plasma levels of FABPs expressed as median and inter-quartile range.

Plasma levels of all 3 FABPs were significantly increased in patients with decompensated cirrhosis compared to those of a group of healthy subjects, while only A-FABP4 plasma levels were significantly higher in patients with decompensated cirrhosis compared to patients with compensated cirrhosis (Fig. 1). In patients with decompensated cirrhosis, no differences were found among plasma levels of FABPs regarding sex or etiology of the disease. The plasma levels of the 3 FABPs showed a positive, albeit weak, correlation among each other. The best correlation was observed between L-FABP1 and I-FABP2 (r = 0.52, p < 0.001).

Table 2 shows FABPs plasma levels of all patients included in the study categorized according to the presence or absence of different complications of cirrhosis. Overall, the presence of any of the major complications of cirrhosis was associated with abnormal FABPs plasma levels. Specifically, increased levels of A-FABP4 were associated with all complications of cirrhosis, except for gastrointestinal bleeding. Results were less consistent with respect to L-FABP1 and I-FABP2.

**Table 2:** Plasma levels of FABPs according to the presence or absence of complications of cirrhosis at inclusion.

	Ascites			Hepatic Encephalopathy			Aki			Bleeding		
	NO n = 91	*p*	YES n = 183	NO n = 186	*p*	YES n = 86	NO n = 146	*p*	YES n = 126	NO n = 225	*p*	YES n = 49
A-FABP4 (ng/mL)	**25** (13–42)	<0.001	**44** (26–87)	**33** (17–61)	0.001	**45** (26–98)	**26** (15–37)	<0.001	**65** (40–109)	**42** (25–83)	<0.001	**21** (13–38)
L-FABP1 (ng/mL)	**22** (14–38)	0.04	**26** (18–40)	**24** (17–37)	0.08	**26** (18–44)	**23** (15–35)	<0.001	**30** (20–45)	**24** (17–37)	0.03	**32** (19–46)
I-FABP2 (ng/mL)	**0.8** (0.4–1.7)	0.001	**1.3** (0.7–2.3)	**1.2** (0.5–2)	0.9	**1** (0.4–2)	**0.9** (0.4–1.8)	0.006	**1.3** (0.7–2.5)	**1.1** (0.5–2)	0.9	**1.1** (0.5–2.2)

FABPs levels are expressed as median (inter-quartile range). A-FABP4: adipocyte fatty-acid binding protein 4, L-FABP1: liver fatty-acid binding protein 1, I-FABP2: intestinal fatty-acid binding protein 2.

Because patients with advanced cirrhosis are prone to develop bacterial infections and A-FABP4 has been proposed as an inflammatory marker, we wanted to assess the relationship between FABPs and bacterial infections in our series of patients. Table 3 shows the univariate analysis of patients with and without infections. As expected, patients with infection had higher levels of blood leukocytes, CRP and plasma IL-6 levels. The presence of infection was associated with high frequency of complications of cirrhosis such as hepatic encephalopathy, acute kidney injury and ACLF as well as more severe liver disease, as indicated by higher MELD and Child-Pugh score values, compared to patients without infection. With respect to FABP, A-FABP4 plasma levels were increased in patients with infection compared to patients without infection, while L-FABP1 and I-FABP2 plasma levels were significantly lower in infected patients. Furthermore, plasma A-FABP4 was the only FABP associated to the development of a new infection during hospitalization, with higher levels of A-FABP4 in those patients that developed an infection during hospitalization compared to those patients that did not developed a new infection [50 (26–107) vs 32 (08–64) ng/mL, respectively p < 0.001]. These differences persisted irrespective of the presence or absence of an infection at inclusion. Among patients with infection, higher plasma levels of A-FABP4 were associated with the presence of shock and ICU admission. On the other hand, no association was found between the type of infection or the use of antibiotic treatment and A-FABP4 levels.

**Table 3:** Characteristics of patients according to the presence or absence of bacterial infection at admission.

	Infected (n = 101)	Non-infected (n = 173)	*p*
Blood leukocytes (10^9^/L)	6.9 (4.5–11.6)	5.4 (3.6–7.8)	0.001
CRP (mg/dL)	3.9 (1.8–6.9)	1.7 (0.7–3.1)	<0.001
IL-6 (pg/mL)	142 (74–272)	89 (45–168)	<0.001
SIRS	51 (51%)	72 (41%)	0.16
Shock	19 (19%)	16 (9%)	0.03
Antibiotic treatment	101 (100%)	57 (33%)	<0.001
MELD score	21 (13–28)	17 (12–22)	0.04
Child-Pugh score	9 (8–12)	9 (7–10)	0.002
MAP (mmHg)	81 (72–88)	83 (76–92)	0.02
Ascites	67 (66%)	116 (67%)	0.9
Hepatic encephalopathy	42 (42%)	44 (25%)	0.004
AKI	56 (55%)	61 (35%)	0.03
ACLF	52 (52%)	61 (35%)	0.011
Development of new infections	21 (21%)	45 (26%)	0.38
A-FABP-4 (ng/mL)	40 (26–89)	36 (18–63)	0.03
L-FABP-1 (ng/mL)	22 (14–37)	27 (18–40)	0.012
I-FABP-2 (ng/mL)	1.3 (0.7–2.1)	0.8 (0.3–1.8)	0.01

Categorical variables are expressed as numbers and percentages (in brackets), continuous variables are expressed as median (inter-quartile range). MELD, model of end stage liver disease; MAP: mean arterial pressure; AKI, acute kidney injury, ACLF: acute-on-chronic liver failure; A-FABP-4: plasma adipocyte fatty acid binding 4; L-FABP-1: plasma liver fatty acid binding 1; I-FABP-2: plasma intestinal fatty acid binding 2.

We next investigated the relationship between plasma levels of FABPs and liver and kidney function, systemic inflammatory parameters, and prognostic scores. L-FABP1 had a weak but positive correlation with AST and ALT (r = 0.26; p < 0.001 for both), but did not correlate with liver function tests, such as bilirubin, INR or albumin. I-FABP2 did not correlate with any of the parameters of liver function. Interestingly, A-FABP4 levels correlated with serum bilirubin (r = 0.3; p < 0.001) and INR (0.36; p < 0.001). There was a significant direct correlation between serum creatinine and plasma levels of FABPs (L-FABP1: r = 0.3, p > 0.001 for; I-FABP2: r = 0.2, p < 0.001 and A-FABP4: r = 0.6, p < 0.001). Finally, only A-FABP4 correlated with cirrhosis severity scores, either Child-Pugh or MELD (r = 0.38, p < 0.001 and r = 0.6, p < 0.001 respectively) (Supplementary Fig. 1). Regarding relationship between plasma levels of FABPs and inflammatory parameters, only A-FABP4 showed a positive correlation with IL-6 plasma levels (r = 0.23, p < 0.001) and leukocytes (r = 0.13; p = 0.03). Interestingly, when infected patients were excluded, A-FABP4 levels positively correlated with plasma IL-6 levels (r = 0.27; p < 0.001) and CRP levels (r = 0.21; p = 0.04).

One-hundred and thirteen out of the 274 patients (41%) had either ACLF at inclusion (95 patients) or developed it during hospitalization (18 patients). As expected, univariate analysis showed that liver and kidney function tests as well as inflammatory parameters were associated with the presence of ACLF (Supplementary Table 1). Plasma levels of FABPs were significantly higher in patients with ACLF compared to those of patients with decompensated cirrhosis without ACLF [A-FABP4: (67 (43–106) vs 26 (15–40 ng/mL p < 0.001; L-FABP1: 30 (20–46) vs 23 (15–34) ng/mL, p < 0.001) and I-FABP2: 1.3 (0.7–2.3) vs 1 (0.4–2) ng/mL, p = 0.039)] (Supplementary Figure 2). Moreover, out of the 3 different FABPs only A-FABP4 was associated with ACLF in multivariate analysis [HR 2.6 (CI 1.2–5.6), p = 0.017].

### Relationship between plasma FABPs and survival

**Figure 2:**
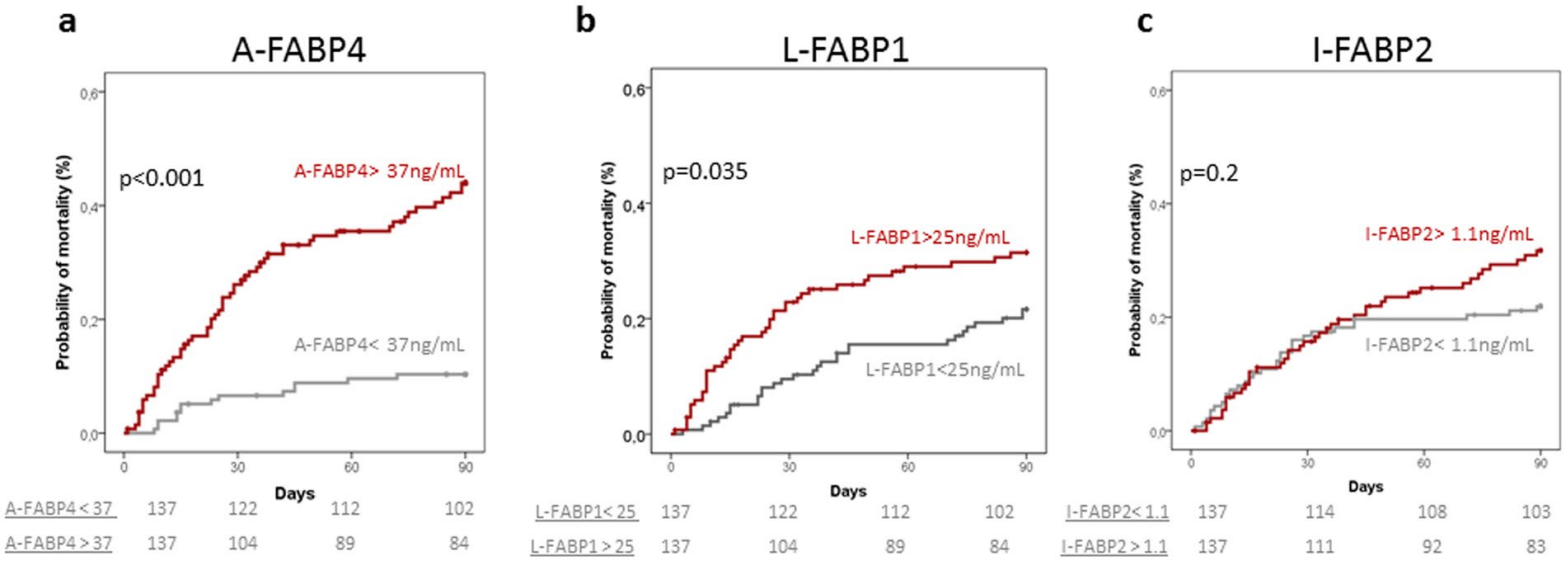
90-day probability of mortality in all patients categorized according to median values of FABPs. A-FABP4: adipocyte fatty-acid binding protein 4, L-FABP1: liver fatty-acid binding protein 1 and I-FABP2: intestinal fatty-acid binding protein 2. Units of median values of FABPs are expressed in ng/mL.

During the 3-month follow-up period, 71 (26%) patients died (44 during hospitalization), 15 were transplanted, and only 1 patient was lost to follow-up. Three-month survival probability in the whole series was 73%. Variables at admission that were associated with 3-month survival in univariate analysis are shown in Table 4. Besides classical prognostic clinical and laboratory variables, plasma levels of A-FABP4 and L-FABP1 were significantly higher in patients who died at three months compared to those of survivors. Figure 2 shows the probability of 3-month mortality of the whole series of patients divided according to median plasma FABPs levels. Both, A-FABP4 and L-FABP1 were associated with mortality. However, in multivariate analysis only A-FABP4 levels were independently associated with 3-month mortality (Table 5). Because there was a relationship between A-FABP4 levels and AKI and infections, we repeated the multivariate analysis excluding patients with AKI and patients with infection. In both cases, the independent prognostic value of plasma A-FABP4 persisted (data not shown), indicating that the prognostic value of A-FABP4 was independent of the impairment of kidney function or presence of infection.

**Table 4:** Characteristics of patients at inclusion in the study according to 90-day survival.

	Alive (n = 203)	Dead (n = 71)	*p*
Age (yr)	58 (52–67)	60 (53–67)	0.896
Gender (Male)	128 (63)	49 (69)	0.391
Chronic kidney disease	33 (16)	12 (16)	0.516
Presence of ascites	123 (61)	60 (84)	<0.001
Presence of encephalopathy	48 (24)	38 (53)	<0.001
Serum bilirubin (mg/dL)	2.0 (1.1–3.4)	5.8 (2.6–22)	<0.001
Serum albumin (g/L)	29 (26–32)	27 (25–32)	0.467
INR	1.5 (1.3–1.8)	1.8 (1.5–2.3)	<0.001
MELD score	16 (12–21)	27 (19–33)	<0.001
Child-Pugh score	8 (7–10)	11 (10–12)	<0.001
Serum creatinine (mg/dL)	1.0 (0.7–1.6)	1.8 (1.0–2.8)	<0.001
Serum sodium (mEq/L)	136 (132–138)	131 (126–136)	<0.001
MAP (mmHg)	83 (77–92)	78 (72–86)	<0.001
Blood leukocytes (10^9^/L)	5.1 (3.5–7.8)	7.8 (5.4–12.6)	<0.001
CRP (mg/dL)	2.0 (0.7–4.8)	2.4 (1.7–6.3)	0.062
AKI	78 (38)	50 (70)	<0.001
ACLF	51 (25)	44 (62)	<0.001
A-FABP-4 (ng/mL)	30 (17–55)	64 (40–106)	<0.001
L-FABP-1 (ng/mL)	24 (16–37)	28 (21–47)	0.005
I-FABP-2 (ng/mL)	1.1 (0.5–2.0)	1.6 (0.6–2.3)	0.2

Categorical variables are expressed as numbers and percentages (in brackets), continuous variables are expressed as median (inter-quartile range). MELD, model of end stage liver disease; MAP: mean arterial pressure; AKI, acute kidney injury, ACLF: acute-on-chronic liver failure; A-FABP-4: plasma adipocyte fatty acid binding 4; L-FABP-1: plasma liver fatty acid binding 1; I-FABP-2: plasma intestinal fatty acid binding 2.

**Table 5:** Cox regression analysis of 90-day transplant-free survival.

Model 1		
Variable	HR (95% CI)	p
Leukocytes (10^9^/L)	2.2 (1.2–2.8)	<0.001
Hepatic Encephalopathy	2.0 (1.2–3.4)	0.02
MELD	1.1 (1.05–1.11)	<0.001
A-FABP4 > 37.2 ng/mL*	2.2 (1.2–4.1)	0.016

HR, hazard ratio; CI, confidence interval; AUROC 0.833.

Variables included in the model: MELD, ascites, hepatic encephalopathy, leukocyte count, L-FABP1, A-FABP4, acute kidney injury, ACLF.

*A-FABP4 was not introduced in the model as a continuous variable because it did not have a normal distribution and there was no linearity between hazard ratios of different quartiles. A categorical distribution with the median value was used instead.

### Hepatic gene expression of FABPs in patients with cirrhosis

**Figure 3:**
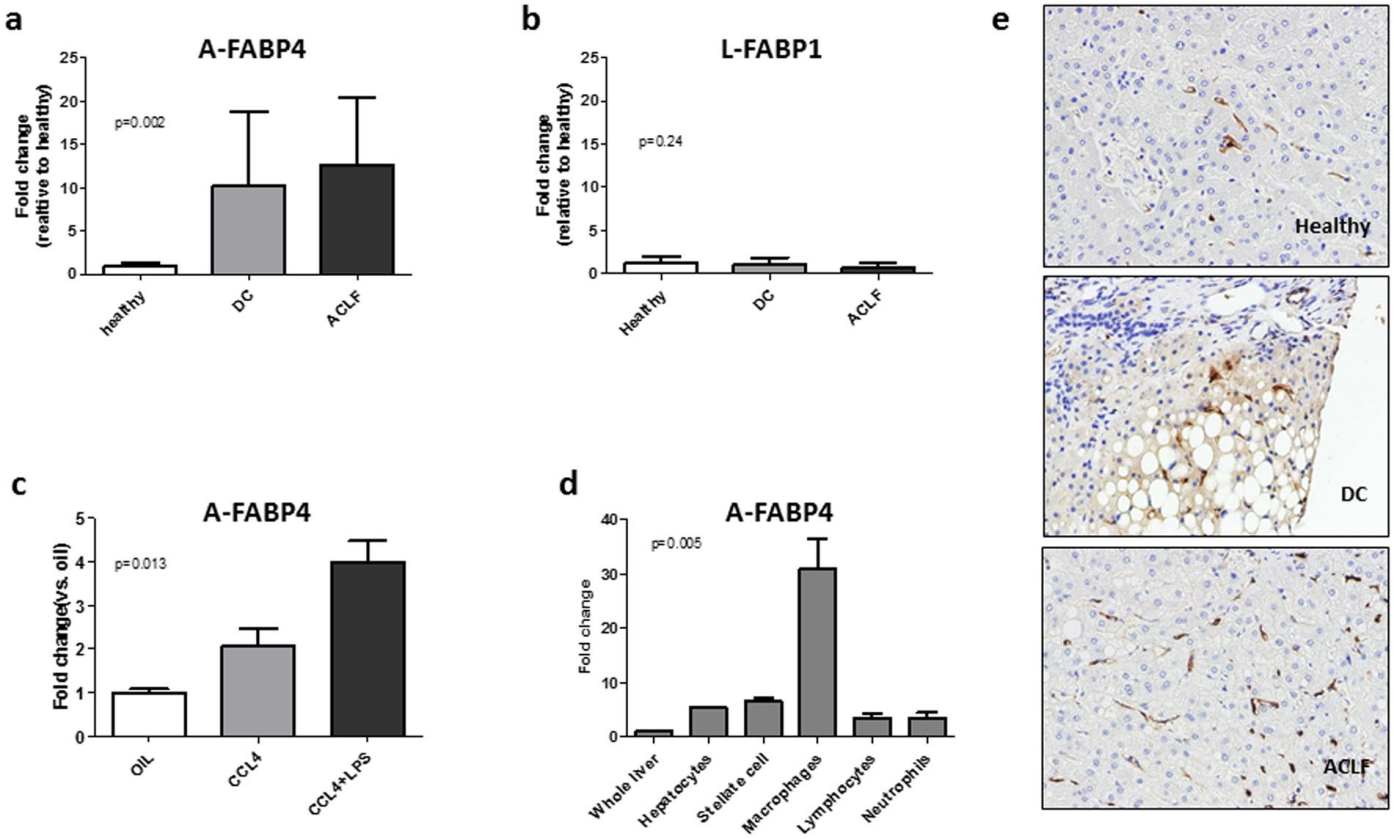
Hepatic gene expression of A-FABP4 and L-FABP1 in patients with decompensated cirrhosis, hepatic A-FABP4 expression in experimental model of acute-on-chronic liver injury and liver cells populations, and immunohistochemistry of A-FABP4 in human liver tissue. Panels a show relative hepatic expression, expressed as fold change, of adipocyte fatty-acid binding protein 4 (A-FABP4) in biopsies of patients with decompensated cirrhosis (n = 12) and ACLF (n = 10). Panel c shows hepatic expression of A-FABP in the whole liver of experimental models of chronic and acute on chronic liver injury (CCl4 and CCL4 + LPS). Panel d shows the expression of A-FABP4 in different liver cells populations in acute on chronic liver injury experimental model (CCL4 + LPS). Panel e shows A-FABP4 staining in liver tissue from a healthy subject, a patient with decompensated cirrhosis and a patient with ACLF. All photos show positive staining for liver macrophages.

We next sought to determine if the liver could be the source of plasma FABPs levels and therefore assess the hepatic gene expression of FABPs. We studied by quantitative real-time PCR the expression of A-FABP4, L-FABP1 and I-FABP2 in liver biopsies from a group of patients with decompensated cirrhosis with and without ACLF (n = 12 and n = 10, respectively). Biopsies from 6 healthy donors obtained at the time of living donor liver transplantation were also analyzed for comparison. As shown in Fig. 3a, hepatic A-FABP4 expression was markedly increased in patients with decompensated cirrhosis, with or without ACLF, compared to that of healthy subjects. By contrast, hepatic L-FABP1 expression was not increased in decompensated cirrhosis, either with or without ACLF (Fig. 3b). I-FABP2 was neither expressed in healthy livers nor in diseased livers.

### Gene expression of A-FABP4 in liver cell populations

Given the increased A-FABP4 gene expression in the liver of patients with decompensated cirrhosis, we next sought to determine which cells were responsible for this increased expression. First, we confirmed in a mouse model of chronic liver disease and acute-on-chronic liver injury that A-FABP4 expression was increased (Fig. 3c). Next, we investigated the expression of A-FABP4 in distinct liver cells subpopulations, including hepatocytes, hepatic stellate cells, macrophages, lymphocytes, and neutrophils from mouse with acute-on-chronic liver injury. Interestingly, among all cell populations explored, macrophages were the only liver cells that markedly and significantly overexpressed A-FABP4 (Fig. 3d). These findings therefore confirm that the increased hepatic expression of A-FABP4 in an experimental model that resembles human disease derives from liver macrophages.

### Immunohistochemistry of A-FABP4 in liver tissue of patients with decompensated cirrhosis

To further explore whether macrophages were the main liver cells expressing A-FABP4 in human disease, we performed immunohistochemistry of A-FABP4 in liver biopsies of patients with decompensated cirrhosis (n = 6). Figure 3e shows that macrophages were positive for A-FABP4 staining in the liver in healthy subjects as well as in patients with decompensated cirrhosis. A-FABP4 staining was increased in macrophages of patients with decompensated cirrhosis and ACLF as compared to healthy subjects.

## Discussion

The results of the current study show that the plasma levels of three different FABPs, A-FABP4, L-FABP1, and I-FABP2, are increased in decompensated cirrhosis with respect to those of healthy subjects and correlate with important disease outcomes. Specifically, among the 3 FABPs analyzed, A-FABP4 plasma levels were those that showed a better correlation with clinical outcomes, specially complications of the disease, presence of ACLF, and short-term mortality. Findings from human liver biopsies demonstrated increased gene expression of A-FABP4, but not L-FABP1 and I-FABP2, in decompensated cirrhosis, which suggests that the increased plasma levels may be, at least in part, from hepatic origin. Finally, gene expression analyses in a mouse model of acute-on-chronic liver injury confirmed the increased hepatic expression of A-FABP4 and provided evidence that the liver macrophages are the cells mainly responsible for the increased hepatic expression, results that were further confirmed by the positive immunohistochemistry for A-FABP4 of macrophages in human diseased livers.

In recent years, evidence has been accumulated indicating that A-FAPB4 is a very versatile protein that has important roles in lipid metabolism, macrophage function, and inflammation^1, 8, 9, 17^. A-FABP4 is one of the most abundant proteins in adipocytes and is an important regulator of lipid metabolism by acting on multiple integrated cellular pathways, involving peroxisome proliferator-activated-ϒ (PPAR-ϒ) and liver X receptor (LXR), to suppress adipose tissue lipogenesis and promote lipolysis. In addition to these effects, A-FAPB4 also participates in inflammation by regulating cycloxigenase-2 activity, promoting leukotriene A4 stability, and acting on a number of other intracellular pathways leading to increased synthesis of inflammatory mediators^9, 18^. Knockout models of A-FABP4 exhibit changes in lipid compositions and metabolism and are resistant to chronic inflammation. On the other hand, expression of A-FABP4 in macrophages is induced during differentiation from monocytes and A-FABP4 is mainly involved in inflammatory responses through activation of the IKK-NF-kB and JNK-AP-1 pathways^1, 9, 19, 20^. Moreover, A-FABP4-deficient macrophages have impaired basal and stimulated expression of inflammatory cytokines, including monocyte-chemoattractant protein-1 (MCP-1) and tumor necrosis factor-α (TNF-α) as well as decreased IKK-NF-kB activity^9^.

Although the main actions of FABPs occur at the intracellular level, many of these proteins reach the extracellular space so that significant levels of FABPs can be detected in plasma. The case of A-FABP4 is particularly remarkable because it has hormone-like effects that include, among others, regulation of hepatic glucose production and immune cell functions^21^. In this regard, increased plasma levels of A-FABP4 have been found in several human diseases, particularly metabolic conditions and inflammatory disorders. A large number of studies have shown that plasma levels of A-FABP4 are increased in obesity and type-2 diabetes mellitus (reviewed in ref. 18). In the Framingham Heart Study it was shown that plasma A-FABP4 levels correlated with increased body mass index, insulin resistance, and dyslipidemia^22^. Other studies have shown that plasma A-FABP4 levels correlate with important clinical outcomes including cardiovascular complications and survival^9^. On the other hand, increased A-FABP4 have also been reported in patients with acute critical illnesses and are associated with poor survival^10^.

The findings of the current study showing that patients with decompensated cirrhosis have increased plasma levels of A-FABP4 that correlate with disease complications, infections, ACLF, and survival are in keeping with those of studies in patients with chronic inflammatory conditions^8, 9^. Findings seem to be quite specific for A-FABP4 and not associated with other members of the FABPs family because correlation with disease outcomes of L-FABP1 or I-FABP2 was less consistent or not existent. The results of our study therefore support the current hypothesis of cirrhosis progression that proposes that both liver and systemic inflammation have a major role in disease progression, from acute decompensation to ACLF, and mortality^11^. Liver inflammation in chronic liver diseases is mainly orchestrated by resident macrophages and infiltrating activated monocytes that promote fibrosis and disease progression^23, 24^. In that regard, it has been shown that plasma markers of macrophage activation, like sCD163 and soluble mannose receptor (sMR), are associated with the presence of ACLF and correlate with prognosis, suggesting and important role of liver macrophages in cirrhosis progression and ACLF development^25^. Interestingly, we found that hepatic expression of A-FABP4 in human liver tissue was increased in patients with decompensated cirrhosis with and without ACLF. Furthermore, liver macrophages from a mouse model of acute on chronic liver injury were the main source of hepatic expression of A-FABP4, findings that were confirmed by the positive A-FABP4 staining of macrophages in patient’s liver tissue. All these findings point towards a role of liver macrophages in driving inflammation and disease progression and ACLF development. With respect to systemic inflammation, increasing evidence suggests that patients with advanced cirrhosis have a marked systemic inflammatory response with increased leukocyte count, C-reactive protein (CRP) levels, and pro-inflammatory cytokines which is independent of the presence of infections and correlates with prognosis^26^. Moreover, it has been shown that this systemic inflammatory reaction, particularly in ACLF, is characterized by alterations in the expression of several cytokines involved in monocyte and macrophage migration^27, 28^. In the current study, plasma A-FABP4 levels correlated with infections and inflammatory parameters and increased levels of A-FABP4 were associated with development and severity of second infection. It is worth noting that the correlation between A-FABP4 plasma levels and plasma IL-6 and CRP persisted in patients without infections. Moreover, A-FABP4 was an independent prognostic factor at multivariate analysis even in non-infected patients. All these data suggest that A-FABP4 levels could be an inflammatory marker of decompensated cirrhosis as it has been shown in critically-ill patients^10^ regardless of the presence or absence of infections. Whether or not A-FABP4 participates in cytokine profile alterations in decompensated cirrhosis is not known, but evidence from A-FABP4-deficient mice showing that there is a reduced expression of cytokines in macrophages makes this hypothesis attractive^29^. The strong association between A-FABP4 levels and mortality found in the present study supports the theory that inflammation is a major driver of disease progression and poor prognosis in cirrhosis.

Although L-FABP1 and I-FABP2 plasma levels were significantly increased in patients with decompensated cirrhosis compared to those of healthy subjects, they showed a much weaker association or no association with disease outcomes compared to A-FABP4. L-FABP1 is a protein that is expressed in the liver and kidney, among other organs. L-FABP1 has been proposed as a marker of hepatocyte injury in acute transplant rejection, but to our knowledge has never been explored in chronic liver diseases. Despite the moderately increased plasma levels, gene expression of L-FABP1 was not increased in the liver of patients with decompensated cirrhosis with or without ACLF. Therefore, it seems that L-FABP1 is not a marker of liver cell injury in advanced cirrhosis. There was no association between I-FABP2 and disease outcomes indicating that plasma levels of intestinal FABP are not a marker of disease severity in cirrhosis.

The current study has some limitations that should be acknowledged. The study was performed in patients with cirrhosis hospitalized for management of an acute decompensation in a tertiary care hospital, with high proportion of patients admitted to ICU (19%). Therefore, whether similar results could be obtained in a less severe population is not known. However, it is important to remark that inclusion of patients with severe liver disease, such as those with ACLF is important to explore the whole spectrum of disease progression. In our study, we only measured FABPs plasma levels in a single time point and unfortunately, we do not have repeated measurements to evaluate whether changes in plasma levels occur over time and bear relationship with disease progression or improvement. This would have to be addressed in future studies. Finally, we measured only 3 out of the 9 FABPs known. We selected those FABPs that we anticipated could have a more prominent role in patients with cirrhosis. Therefore, we do not know whether other FABPs could be related to disease outcomes in cirrhosis.

In summary, the results of the current study indicate that plasma A-FABP4 levels are increased in decompensated cirrhosis and correlate with hard clinical outcomes, specifically complications of the disease, ACLF, and mortality. Results from gene expression in the liver indicate that A-FABP4, but not the liver-associated type L-FABP1, is increased in the cirrhotic liver tissue and liver macrophages appear to be the cell type responsible for this increased gene expression. Our results are consistent with a major role of inflammation in cirrhosis progression and clinical outcomes.

## Methods

### Study population

This is a prospective study of 274 consecutive patients with cirrhosis hospitalized for management of an acute decompensation of the disease at the Liver Unit of Hospital Clinic of Barcelona. These patients were included in a prospective investigation assessing mechanistic factors and markers of disease progression in cirrhosis between 2009 and 2012. Patients had their clinical information collected into a specific database and plasma and urine samples were taken at admission and stored into a biobank (*Biobanco Hepatorenal R11-0602-054*). All consecutive patients with cirrhosis admitted to the hospital were included in the biobank database except for the following: 1/patients with hepatocellular carcinoma outside the Milan criteria; 2/patients admitted to the day hospital for elective diagnosis or therapeutic procedures; 3/patients previously treated with liver and/or kidney transplantation; 4/patients on chronic hemodialysis; and 5/refusal of informed consent. All patients gave written informed consent for data and sample collection and analytical assessment. The protocol was approved by the Institutional Review Board of the Hospital Clinic of Barcelona and all experiments were performed in accordance with relevant guidelines and regulations. A group of 13 patients with compensated cirrhosis and 13 healthy subjects were also studied for comparison.

The diagnosis of cirrhosis was made using standard criteria either with liver biopsy or combination of clinical, analytical, and ultrasonographic findings. The diagnosis of ascites, hepatic encephalopathy, bacterial infections, gastrointestinal bleeding, and acute-on-chronic liver failure (ACLF) was made using criteria reported elsewhere^26, 30–32^. Acute impairment of kidney function (AKI) was defined using the AKIN criteria adapted to patients with cirrhosis^33, 34^. During admission, complications of cirrhosis were managed according to international guidelines^30, 32, 33^.

### Plasma Fatty-acid binding protein measurements

Plasma samples were obtained in the morning, approximately at 9 a.m., and centrifuged at 2000 rpm for 10 minutes and the supernatant was stored at −80 °C until analysis. L-FABP1, I-FABP2 and A-FABP4 were measured with the human L-FABP1 ELISA kit (Hycult Biotech; Uden, The Neatherlands), the human I-FABP2 ELISA kit (Hycult Biotech; Uden, The Neatherlands) and the Quantikine ELISA human A-FABP4 kit (R&D Systems, Minneapolis, USA), respectively. Interleukin-6 (IL-6) was measured with human ELISA IL-6 kit (Diasource; Louvain-la-Neuve, Belgium). Coefficients of inter-assay and intra-assay variation for plasma FABPs were lower than 10 and 15%, respectively.

### Hepatic Gene expression of FABPs

#### Mouse models of liver injury

Mice aged 8–10 weeks were subjected to a chronic liver injury model by injecting carbon tetrachloride (CCl_4_) intraperitoneally (Sigma-Aldrich; diluted 1:4 in corn oil) at dose of 0.5 ml/kg body weight twice per week for a total of 5 injections (n = 4). Control mice (n = 3) were given vehicle (corn oil, Sigma- Aldrich). We also performed a model of acute-on-chronic liver injury by the administration of lipopolysaccharide (LPS, Sigma-Aldrich) 10 mg/kg on the chronic damage caused by CCl_4_. Animal procedures complied with the institution’s guidelines, received human care and were approved by the ethics committee of the University of Barcelona and all experiments were performed in accordance with relevant guidelines and regulations.

#### Murine hepatic cell sorting

Different hepatic cell populations were isolated from the livers of mice treated with CCl_4_ and LPS. Cells were isolated as previously described^35^. Briefly, after two-step collagenase-pronase perfusion of livers followed by Nycodenz density gradient centrifugation, obtained cells were incubated with CD3- Alexa Fluor 700 (T cells), F4/80-Alexa Fluor 647 (Serotec) (macrophages), Ly6G-APC (eBioscience, Affymetrix, San Diego, CA, USA) (neutrophils), and HSC were purified by vitamin A-based flow cytometry. All samples were purified by high-speed sorting using a FACS-Aria cell sorter (Becton, Dickinson and Company, BD, New Jersey, NJ, USA).

#### mRNA isolation and gene expression assay

mRNA was isolated from human and murine liver tissues using Trizol and following manufacturer’s manual instructions (Invitrogen, Carlsbad, CA, USA). mRNA from sorted cells was harvested by using the QIAGEN RNeasy MICROKit (QIAGEN GmbH, Hilden, Germany). mRNA levels were evaluated by quantitative real time PCR on an ABI 7900HT cycler (Applied Biosystems) using Taqman gene expression assays (Life technologies). Individual gene expression was normalized to 18 s or GAPDH. Relative expression was calculated using the comparative Ct method (2^−ΔΔCt^).

### Immunohistochemistry

Human paraffin embedded liver sections were used for immunohistochemistry using the Leica Microsystems’ Bond-Max™ automated immunostainer together with the Bond Polymer Refine Detection System (Leica Microsystems, Spain). Briefly, samples were deparaffinized and antigen retrieval performed in citrate buffer (pH 6, 20 minutes). Sections were then immunostained with primary antibody to FABP4 (dilution 1:1500, Abcam, UK) for 1 hour at room temperature, developed with diaminobenzidine and counterstained with hematoxylin.

### Statistical analysis

Results for continuous variables were expressed as median and interquartile range. Counts and percentages were used for the description of the categorical variables. Comparisons between two independent groups were made with the t-test (previously checking the hypothesis of variance homogeneity) for continuous normal-distributed variables. The Mann-Whitney U test was carried out for continuous non-normal distributed variables in the case of 2 independent groups, whereas the Kruskal-Wallis test was performed when comparing a higher number of groups. Comparisons of categorical variables among groups were made with chi-squared test or Fisher test if appropriate.

Survival analyses were performed with Kaplan-Meier curves and long-rank test. Multivariate logistic regression models were fitted with all variables that were predictive of ACLF at univariate analysis to select main factors independently associated with ACLF. Multivariate cox-regression analysis was performed to investigate independent predictor factors of three-month survival. Variables with skewed distribution were log-transformed before being included in the multivariate analysis model. Regarding plasma A-FABP4 levels, the variable followed a non-normal distribution and the linearity was lacking, being the risk for the fourth quartile, if not less than for third quartile, at least is quite similar to it. Therefore, in order not to overestimate the risk for the last quartile we categorized the variable as median to include in the cox-regression analysis. The significance level for all statistical tests was set at 0.05 two-tailed. All statistical analyses were performed using SPSS 20.0 software.

## Electronic supplementary material

Supplementary material
