# Supplementary material for: Adipocyte Fatty-Acid Binding Protein is Overexpressed in Cirrhosis and Correlates with Clinical Outcomes
Source: Sci Rep. 2017 May 12;7:1829. doi: 10.1038/s41598-017-01709-0 (PMC5431836; doi:10.1038/s41598-017-01709-0)
Supplement: Supplementary file 1 — Supplementary material [file 41598_2017_1709_MOESM1_ESM.pdf]

ADIPOCYTE FATTY-ACID BINDING PROTEIN IS OVEREXPRESSED IN CIRRHOSIS AND CORRELATES WITH CLINICAL OUTCOMES

Isabel Graupera[1,2,3], Mar Coll[2,3], Elisa Pose[1,2], Chiara Elia[1], Salvatore Piano[1], Elsa Solà[1,2,3,4], Delia Blaya[2], Patricia Huelin[1,2,3], Cristina Solé[1,2,3], Rebeca Moreira[1,2,3], Gloria de Prada[1], Núria Fabrellas[2,4], Adrià Juanola[1] , Manuel Morales-Ruiz[2,3,5], Pau Sancho-Bru[2,3], Càndid Villanueva[3,6] and Pere Ginès*[1,2,3,4].

[1]Liver Unit, Hospital Clinic, University of Barcelona, Barcelona, Spain.

[2]Institut d'Investigacions Biomèdiques August Pi I Sunyer (IDIBAPS), Barcelona, Spain.

[3]Centro de Investigación Biomédica en Red de Enfermedades Hepáticas y Digestivas (CIBEREHD).

[4]School of Medicine and Health Sciences Center, University of Barcelona, Barcelona, Spain

[5]Biochemistry and Molecular Genetics Department. Hospital Clínic, Department of Biomedicine-Biochemistry Unit, School of Medicine University of Barcelona, Barcelona, Spain.

[6]Gastroenterology department, Hospital de la Santa Creu i Sant Pau, Barcelona, Spain.

***Corresponding author:**

Dr Pere Ginès

LiverUnit

Hospital Clinic de Barcelona

Barcelona

Spain

Phone number: 0034932271713

Email: pgines@clinic.cat

Supplementary Table 1. Characteristics of patients according to the presence or absence of ACLF

	No ACLF (n=161)	ACLF (n=113)	*p*
Age (yr)	60 (52-70)	58 (52-65)	0.069
Gender (Male)	96 (60)	81 (72)	0.041
Etiology of cirrhosis: Alcoholic/ Hepatitis C/ Other – n (%)	82/71/8	67/44/2	0.45
Chronic kidney disease	11(7)	34 (30)	<0.001
Presence of ascites	86 (53%)	97 (86%)	<0.001
Presence of encephalopathy	34 (21%)	52 (47%)	<0.001
Serum bilirubin (mg/dL)	2 (1.1-3.2)	4.6 (1.9-16)	<0.001
Serum albumin (g/L)	28 (25-32)	29 (25-32)	0.606
INR	1.4 (1.2-1.6)	1.8 (1.5-2.3)	<0.001
MELD score	14 (11-17)	27 (22-31)	<0.001
Child-Pugh score	8 (7-10)	10 (9-12)	<0.001
Serum Creatinine (mg/dL)	0.9 (0.7-1.1)	2 (1.5-2.9)	<0.001
Serum sodium (mEq/L)	136 (133-138)	133 (126-136)	<0.001
MAP (mmHg)	84 (78-94)	27 (22-31)	<0.001
Leukocytes (10^9 /L)	5.1 (3.7-7.7)	6.8 (4.7-10.7)	0.001
CRP (mg/dL)	1.8 (0.7-3.6)	2.9 (1.7-5.9)	0.003
pl A-FABP-4 (ng/mL)	26 (15-40)	67 (43-107)	<0.001
pl L-FABP-1 (ng/mL)	23 (15-34)	30 (20-46)	<0.001
pl I-FABP-2 (ng/mL)	1.0 (0.4-2.3)	1.3 (0.7-2.3)	0.040

Legend: Categorical variables are expressed as numbers and percentages (in brackets), continuous variables are expressed as median (inter-quartile range). MELD, model of end stage liver disease; pl A-FABP-4: plasma adipocyte fatty acid binding 4; pl L-FABP-1: plasma liver fatty acid binding 1; pl I-FABP-2: plasma intestinal fatty acid binding 2.

SUPPLEMENTARY FIGURE 1

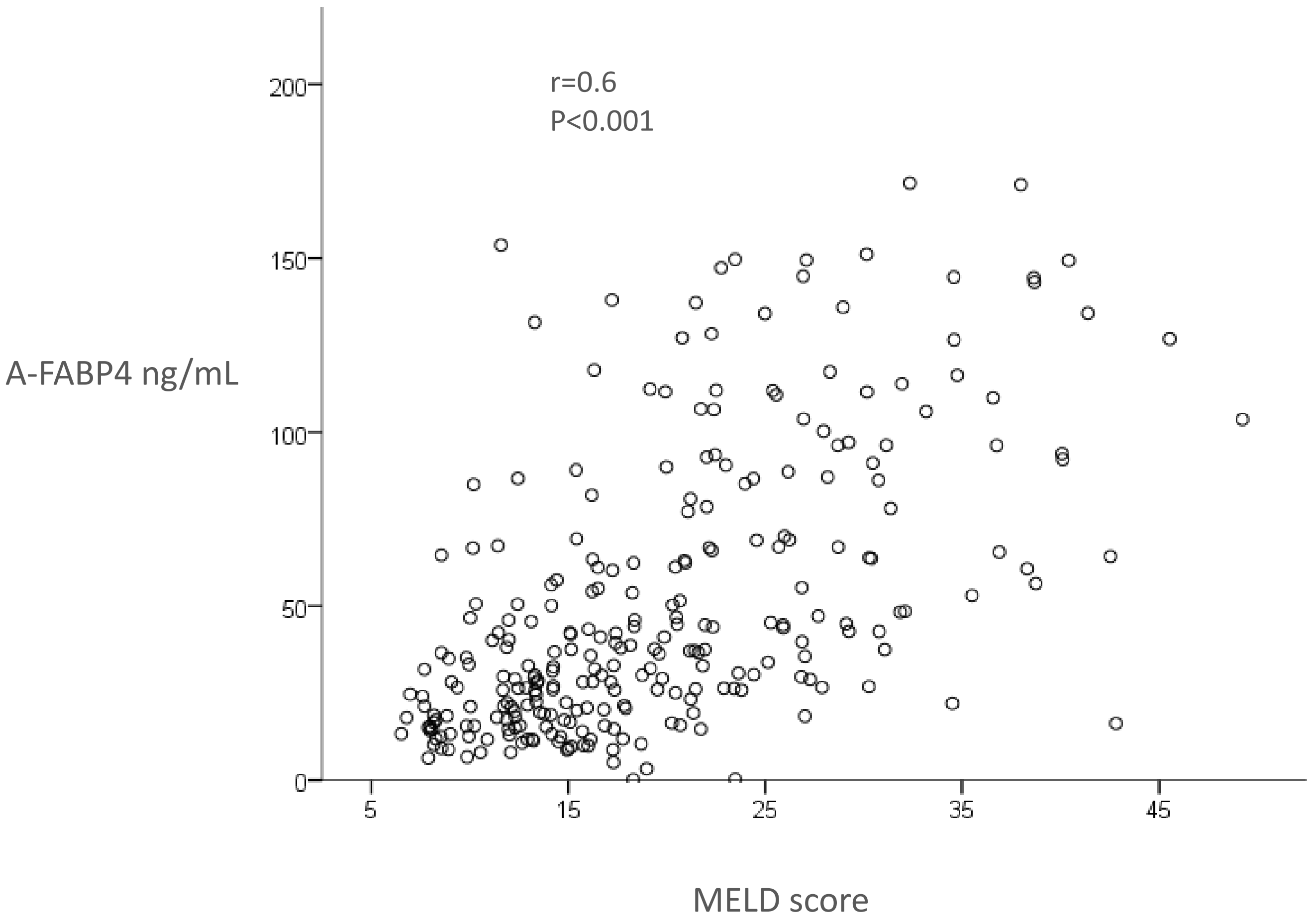

Supplementary Fig 1. Correlation between plasma levels of A-FABP4 and MELD score. A-FABP4: adipocyte fatty-acid binding protein 4, MELD: model for end-stage liver disease. Units for A-FABP4 are ng/mL.

Supplementary Fig 2. Plasma levels of FABPs in patients with decompensated cirrhosis according to the presence or absence of ACLF. Panel A shows plasma levels of adipocyte fatty-acid binding protein 4 (A-FABP4), panel B shows plasma levels of liver fatty-acid binding protein 1 (L-FABP1), and panel C shows plasma levels of intestinal fatty-acid binding protein 2 (I-FABP2). Values shown below the whisker-plots are plasma levels of FABPs expressed as median and inter-quartile range. Patients without ACLF n= 161, and patients with ACLF n= 113.

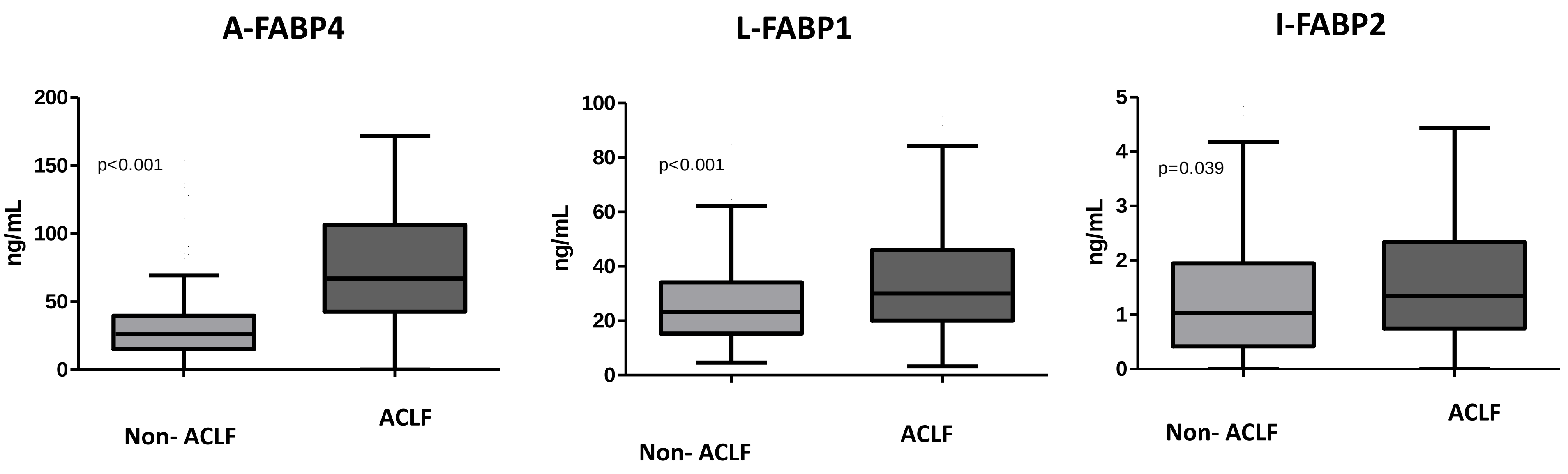

26 (15-40) **67** (43-106)

23 (15-34) **30** (20-46)

1 (0.4-2) **1.3** (0.7-2.3)

SUPPLEMENTARY FIGURE 2
